# Phase I/II trial investigating gedatolisib plus talazoparib in advanced triple negative or BRCA1/2 positive, HER2 negative breast cancers

**DOI:** 10.1007/s10549-025-07747-x

**Published:** 2025-06-05

**Authors:** Sneha Phadke, Kathy D. Miller, Ami Shah, Oana C. Danciu, Yi Chen, Menggang Yu, Mark E. Burkard, Kari B. Wisinski

**Affiliations:** 1 Carver College of Medicine Department of Internal Medicine, University of Iowa, 200 Hawkins Drive, C21GH, Iowa City, IA 52242 USA; 2 Melvin and Bren Simon Comprehensive Cancer Center, Indiana University, Indianapolis, IN USA; 3 Robert H Lurie Comprehensive Cancer Center, Northwestern University, Chicago, IL USA; 4 University of Illinois Cancer Center, Chicago, IL USA; 5 Carbone Cancer Center, University of Wisconsin-Madison, Madison, WI USA; 6 Department of Medicine, University of Wisconsin-Madison, Madison, WI USA; 7 Department of Biostatistics and Medical Informatics, University of Wisconsin-Madison, Madison, WI USA; 8 Department of Biostatistics, University of Michigan - Ann Arbor, Ann Arbor, MI USA

**Keywords:** PARP inhibitor, MTOR/PI3 K inhibitor, Triple negative breast cancer, Homologous repair deficiency

## Abstract

**Purpose:**

Metastatic triple negative breast cancer has a poor prognosis with limited targeted treatment options. In preclinical studies PI3K inhibition led to increased DNA damage and subsequent sensitization to PARP inhibition. This study aimed to investigate the safety and efficacy of the combination of an mTOR/pan-PI3K inhibitor, gedatolisib, with the PARP inhibitor, talazoparib, in patients with advanced triple negative breast cancer or advanced HER2 negative breast cancer and a germline BRCA1/2 mutation.

**Methods:**

The primary objective of the safety run-in was safety and tolerability of the combination and for dose escalation to find the maximum tolerated dose. A 3 + 3 design was utilized for dose escalation. The primary objective of the phase II study was objective response rate (ORR) in the patients with wildtype germline BRCA1/2. The prespecified efficacy threshold was 20%. Secondary objectives included progression-free (PFS) and overall survival (OS) as well as correlative testing, examining homologous recombination deficiency (HRD) status.

**Results:**

The combination of gedatolisib and talazoparib carried manageable toxicities with a low incidence of grade 3 adverse events. The most common adverse events of all grades were anemia, fatigue, and oral mucositis. The ORR in the phase II study was 12%. There were no new safety signals identified in the phase II study. mPFS was 2.5 months (95% CI 1.71, 9.89), and mOS was 7 months (95% CI 4.3, NA) in the full phase II cohort. HRD status was analyzed by high (≥ 33) or low (< 33) genomic instability score, and there was no difference in response rate between the groups.

**Conclusion:**

The combination of gedatolisib and talazoparib is safe but did not meet the prespecified efficacy threshold for objective response rate. Additional preclinical studies of these pathways are warranted prior to future clinical trials of the combination.

Trial Registration: ClinicalTrials.gov ID: NCT03911973, Date of registration: 2019-04-11.

**Supplementary Information:**

The online version contains supplementary material available at 10.1007/s10549-025-07747-x.

## Background

Patients with triple negative breast cancer (TNBC) have fewer targeted options for treatment of metastatic disease than other breast cancer subtypes. This aggressive breast cancer subtype also has the lowest overall survival rate among advanced breast cancers, with a median survival of 12–13 months [1, 2]. Although immune checkpoint inhibitors and antibody drug conjugates have emerged as new therapies, these approaches still include chemotherapy and its associated side effects and have diminishing efficacy in later lines of therapy. A subset of breast cancer patients harbor a germline mutation in BRCA1/2 (gBRCA1/2 m), associated with defects in homologous recombination (HR) DNA repair. Poly(ADP-ribose) polymerase (PARP) enzymes are involved in DNA repair and are activated by DNA strand breaks, making them particularly critical in these BRCA1/2-deficient tumors [3, 4]. PARP inhibitors (PARPi) are now standard therapeutic agents for patients with HER2-negative advanced breast cancer and a gBRCA1/2 m based on phase III trials showing improvement in progression-free survival (PFS) [5, 6]. Whether PARP inhibitors can be extended to other cohorts with TNBC remains a key question.

Approximately 30–50% of breast cancer patients have a somatic alteration in the PI3 K/AKT/mTOR signaling pathway, driving tumor growth, proliferation, metabolism, and survival [7, 8]. Independent of these pathway alterations, one key function of PI3 K involves stabilizing double-strand break repair by interacting with the HR complex [9, 10]. One pre-clinical study showed that PI3 K inhibition in BRCA-proficient TNBC tumor xenografts led to increased DNA damage and subsequent sensitization to PARP inhibition [11]. Other preclinical data suggests that PI3 K inhibitors (PI3 Ki) enhance the efficacy of PARPi by inhibiting the detection and repair of double-strand breaks, by increasing anti-metabolic activity, and by lowering nucleotide pools that are required for DNA synthesis and S-phase progression. [12, 13].

A phase I study evaluating the PI3 Ki, buparlisib, with olaparib in patients with ovarian and breast cancer showed that the agents can be safely used in combination, with responses seen in those with triple negative breast cancer and both mutant and wildtype gBRCA1/2 [14]. Alpelisib and olaparib were combined in another phase I trial evaluating safety in patients with wildtype or gBRCA1/2 m advanced ovarian cancer, with results showing the combination to be safe with preliminary evidence of efficacy, including in those patients with wildtype BRCA1/2 [15]. Based on this preclinical and clinical data, we hypothesized that combination therapy with a dual mTOR/pan-PI3 K inhibitor, gedatolisib, and the PARPi, talazoparib, may be effective in breast cancer patients with a gBRCA1/2 m and in patients with BRCA1/2-wildtype TNBC.

Some patients with wildtype BRCA1/2 may have other defects in the HR pathway, leading to tumors that display a “BRCA-ness"phenotype [16]. The Olaparib Extended study evaluated olaparib in patients with metastatic breast cancer and somatic BRCA1/2 mutations (sBRCA1/2 m) or with germline or somatic mutations in HR-related genes other than BRCA1/2. Results of the study revealed a 50% objective response rate (ORR) and 13.3 month PFS in the patients with a sBRCA1/2 m [17]. Several assays are available that can quantify homologous recombination deficiency (HRD). One such assay quantifies 3 different measures of genomic instability (GIS) including loss of heterozygosity, telomeric allelic imbalance, and large-scale state transitions [18]. A high GIS (≥ 42) was identified in patients with early stage TNBC that was responsive to platinum chemotherapy [19, 20]. Other analyses indicated that a cutoff of ≥ 33 was a better predictor of response in a cohort of patients with ovarian cancer or TNBC [21, 22]. We explored HRD as a potential biomarker of response in the phase II TNBC cohort with wildtype BRCA1/2.

## Methods

### Study design and oversight

**Fig. 1:**
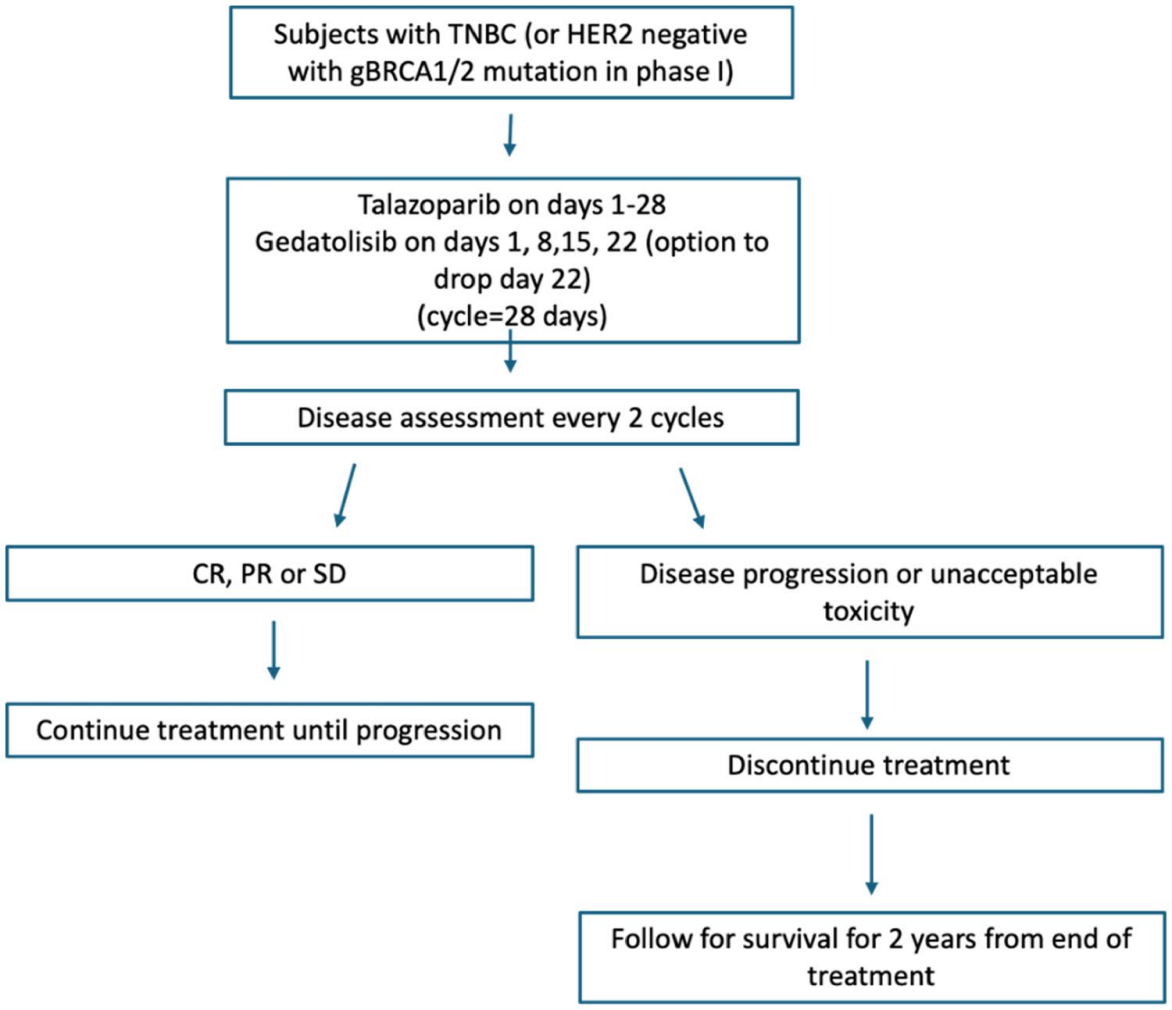
Trail schema

This trial was designed as a non-randomized open-label study. Informed consent was obtained from all individual participants included in the study. We conducted an initial safety run-in phase with a primary objective to evaluate dose limiting toxicity (DLT) and determine recommended phase II doses (RP2D) of talazoparib in combination with gedatolisib. Given that these agents did not have known overlapping DLTs from the single-agent phase I studies, a safety run-in was planned, instead of completing a formal phase I trial. Figure 1 shows the trial schema. A 3 + 3 design was utilized for dose escalation, requiring between 9 and 18 patients, with starting dose at level 1 (0.75 mg oral daily talazoparib and 180 mg IV weekly of gedatolisib) with 28-day cycles. The MTD was the highest explored dose of talazoparib combined with gedatolisib at which ≤ 1 out of 6 subjects experienced a DLT within the first cycle of therapy. The maximum tolerated dose (MTD) was planned as the recommended Phase II dose (RP2D).

Patients evaluable for response had measurable disease at baseline, received ≥ 1 cycles of therapy and had at least one imaging assessment per protocol, unless they were deemed to have rapid clinical progression prior to imaging. For phase II patients who were removed from the study due to concern for clinical progression prior to the first imaging assessment, there was discussion between the principal investigators and treating physicians to confirm agreement in the determination of clinical progression without imaging. If clinical progression was determined in the absence of imaging, best tumor response was classified as progressive disease.

Any subject who received ≥ 1 dose of treatment was evaluable for toxicity, and in the safety run-in, any patient who completed cycle 1 of therapy was evaluable for DLT. ORR was estimated by dividing the total number of responders by the number of subjects with measurable disease.

The phase II trial then began once the RP2D was established, with a primary objective to estimate the ORR [(including confirmed complete responses (CR) and partial responses (PR)] in patients with BRCA1/2 wildtype (or BRCA1/2 unknown) metastatic TNBC (mTNBC). The sample size was determined based on a one-sided binomial test for the null (5%) and alternative (20%) ORR. The recommendation or rejection boundary was 4; therefore if 4 or more responses were observed, the treatment would be recommended. With type I error rate of 0.1 and a power level of 80%, 17 subjects were needed for the phase II study. The number of patients accrued was set at 18 so that we could account for dropout, unevaluable, or missing outcomes.

Secondary objectives included estimating the duration of response (DoR), clinical benefit rate (CBR), PFS, and overall survival (OS). DoR was defined as the number of months from the start date of the confirmed CR or PR, whichever was observed first, to the first date that progressive disease (PD) was documented. The date of all-cause death was used as the response end date if the patient did not have documentation of PD beforehand. The CBR was reported at 16 weeks [including CR, PR, or stable disease (SD)]. PFS was defined as time from the date of treatment initiation to the date of recurrence or death from any cause whichever occurred first. OS was defined by the date of treatment initiation to date of death from any cause. Subjects who had not died were right censored at the date of the last follow-up. We used the Kaplan–Meier method to graphically display survival probabilities and compute median PFS and OS with associated 95% confidence intervals. All statistical tests were two-sided, and 5% was used as the level of significance. Statistical analysis was done in R 4.4.0, including the “survival” and “survminer” packages.

### Participants and treatment

Patients were recruited from 5 academic cancer centers through the Big Ten Cancer Research Consortium (BTCRC). Patients were eligible for participation in the trial if they had advanced HER2 negative breast cancer that had progressed on up to 2 lines of prior therapy (later amended to include up to 3 prior lines given new drug approvals in mTNBC). Patients with hormone-receptor positive breast cancer were eligible for the safety run-in if they had a gBRCA1/2 m. Protocol therapy was discontinued at the time of disease progression, by physician or participant request, unacceptable therapy-related toxicity, or if protocol therapy was interrupted for > 21 days. Subjects were registered through the BTCRC Administrative Headquarters electronic data capture system and started protocol therapy within 7 days of registration.

In the safety run-in phase, patients received gedatolisib 150–180 mg IV on days 1, 8, 15, and 22 and talazoparib (0.75-1 mg) in escalated doses, oral, once daily in a 28-day cycle. Dexamethasone oral rinse was recommended for prevention of oral mucositis. A protocol amendment allowed the phase II cohort to proceed with a 3 week on/1 week off regimen of gedatolisib, as new data emerged suggesting enhanced efficacy and a more favorable safety profile with this dosing schema [23]. The dose levels in the safety run-in are detailed in Supplementary Table S1. In the phase II trial, there were 2 cohorts initially planned, with Cohort A enrolling patients with advanced TNBC (defined as estrogen receptor (ER) and progesterone receptor (PR) < 10%) and Cohort B, as an exploratory cohort, enrolling patients with advanced HER2 negative breast cancer and a gBRCA1/2 m. The protocol was later amended, closing Cohort B early due to changes in prioritization and funding, and patients with a gBRCA1/2 m were then excluded from participation in the trial.

### Assessments

Imaging assessment with computed tomography (CT) of the chest, CT or MRI of the abdomen, and pelvis was mandatory at screening, every 2 cycles thereafter until 6 months and then could be extended to every 3 cycles, and then 30-days post-last dose of protocol therapy. Bone or PET scan was mandatory at screening and then every 4 cycles thereafter, if bone metastases were present. Brain imaging was mandatory at screening and then as clinically indicated.

Fasting lipid panel and hemoglobin A1c were done at baseline, after cycle 1, and then every 3 cycles thereafter. All treatment-emergent adverse events (TEAEs) were recorded using National Cancer Institute Common Terminology Criteria for Adverse Events (NCI CTCAE) version 5.

## Results

### Patients

**Fig. 2:**
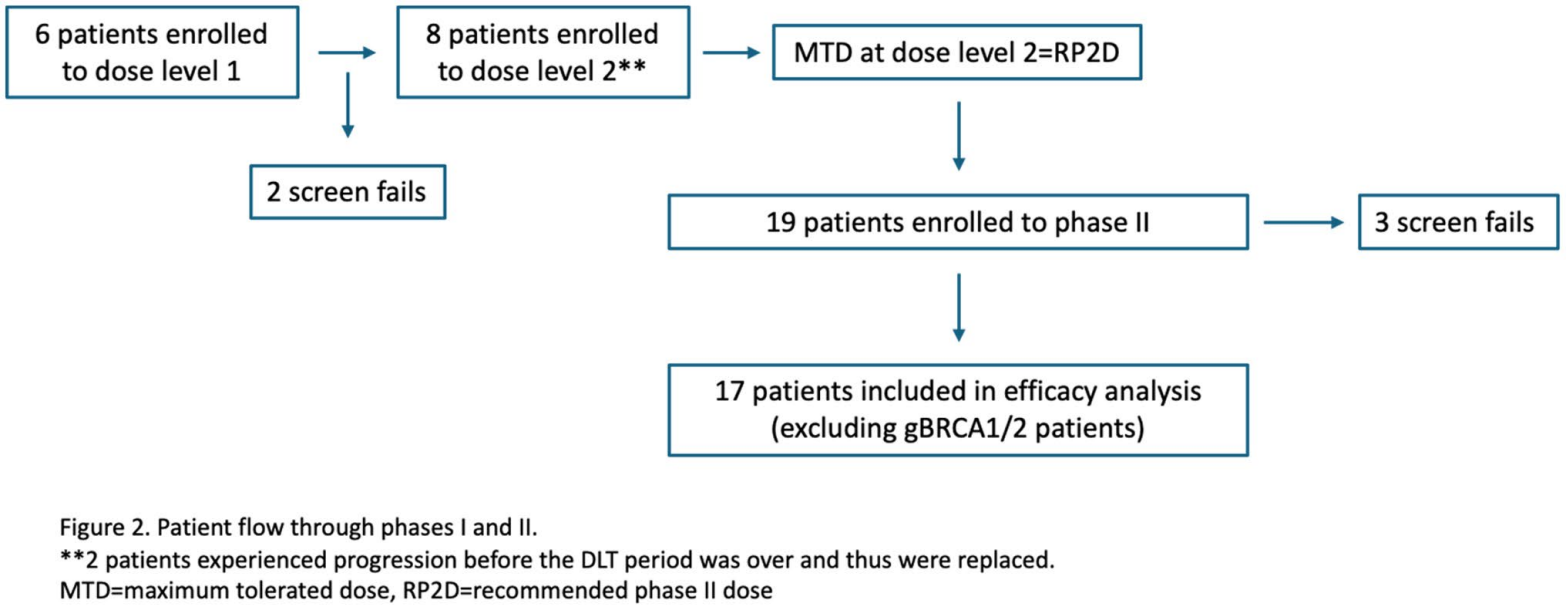
Patient flow through phases I and II. **2 patients experienced progression before the DLT period was over and thus were replaced. *MTD* maximum tolerated dose, *RP2D* recommended phase II dose

From 06/05/19–05/03/21, the safety run-in enrolled 14 female patients. Median age was 53 (range 30–67). One patient had a gBRCA1 m, 1 had a gBRCA2 m, and 1 patient had a sBRCA1 m in the tumor but did not have a germline mutation. Patients had a median of 1.5 prior lines of therapy (range 0–2). The phase II cohorts enrolled 19 female patients from 08/06/21–10/04/22, with a median age of 50 (range 32–79) and median of 1 line of prior therapy (range 0–3). Two patients with a gBRCA2 m were enrolled; 1 patient enrolled to cohort B prior to the amendment that excluded gBRCA1/2 m patients and the other was found to have a gBRCA2 m after enrollment in the trial and was enrolled to cohort A as unknown. The demographic and clinical characteristics of all enrolled patients are summarized in Table 1. Figure 2 depicts patient flow through both phases of the trial.

**Table 1:** Baseline patient characteristics

Patient Characteristics	Phase I (*n* = 14)		Phase II (*n* = 19)	
Median age	53 (30–67)		50 (32–79)	
Gender	14, 100% female		19, 100% female	
Race	Black/African American	2 (14%)	Asian	1 (5.3%)
	White	11 (79%)	White	17 (89%)
	Unknown	1 (7.1%)	Native American/Alaskan native	1 (5.3%)
Median number of prior lines of therapy for metastatic disease	1.5 (range 0–2)		1 (range 0–3)	
Germline *BRCA* mutation	*BRCA1*	2	*BRCA1*	0
	*BRCA2*	1	*BRCA2*	2
Receptor subtype	ER positive**	2	ER positive***	1
	ER negative^	12	ER negative	18

^**^One patient in the phase I had wildtype BRCA½ with ER + disease but met eligibility with < 10% ER expression. A second patient was enrolled with > 10% expression based on report of a second biopsy that was ER negative, however, this was unconfirmed

^Two patients were positive for PR, one that was 3% positive and met eligibility. The other was 30% PR positive. As this patient was in the phase I and efficacy was not analyzed in this cohort, this patient’s safety data was retained

^***^The patient with ER + disease also had a germline BRCA2 mutation and was enrolled in the original Cohort B, which was subsequently closed. This pt was excluded from the primary analysis on response

### Phase I/dose escalation

There were 6 patients enrolled to dose level 1 and received intended treatment. One patient enrolled with a history of a prior biopsy that was ER 20% positive. There was report of a subsequent biopsy that was ER negative, but this was unconfirmed. Another patient enrolled with ER negative but PR 30% positive breast cancer. Both discrepancies were discovered after the patients had completed the dose escalation/phase I trial. There was no statistical analysis for response done for this cohort, and the data from these 2 patients were included in the safety analysis.

There was 1 DLT of grade 3 neutropenia at dose level 1. There were 8 patients enrolled to dose level 2 as 2 patients experienced progression before the DLT period was over and thus were classified as inevaluable per protocol and replaced. There was 1 DLT of thrombocytopenia at dose level 2. Three patients experienced grade 4 AEs (2 thrombocytopenia and 1 lymphopenia), which were outside the DLT window. The MTD was confirmed as 1 mg talazoparib daily and 180 mg gedatolisib weekly, which were selected as the RP2D. The median duration of study therapy was 2.18 months (range 0.23–39.23 months).

### Safety

The safety analysis included all patients in the phase I and phase II trials who had received at least 1 dose of study therapy. Most treatment-emergent AEs were grade 1–2. The most common drug-related AEs are detailed in Table 2. There were 9 patients who developed hyperglycemia, 1 of whom had grade 3 hyperglycemia. Grade 3 AEs consisted mostly of myelosuppression. There were 2 patients who experienced grade 3 pneumonitis. One of those patients was categorized as experiencing a serious AE (SAE) requiring hospitalization. Both patients who developed grade 3 pneumonitis discontinued study therapy due to this AE, and both instances were attributed to gedatolisib. There were 2 additional cases of pneumonitis that were grade 2, also attributed to gedatolisib. Four patients discontinued study therapy due to grade 3 AEs of mucositis, pneumonitis, fatigue, and neutropenia.

**Table 2:** Treatment-emergent adverse events

Most common AE	All grades (*n* = 33)	Grade 1	Grade 2	Grade 3	Grade 4
Anemia	23 (70%)	6 (26%)	8 (35%)	8 (35%)	1 (4%)
Fatigue	23 (70%)	12 (52%)	6 (26%)	5 (22%)	0
Oral mucositis	21 (64%)	8 (38%)	11 (52%)	2 (10%)	0
Nausea	21 (61%)	14 (67%)	7 (33%)	0	0
Neutropenia	15 (45%)	3 (20%)	9 (60%)	3 (20%)	0
Anorexia	15 (45%)	9 (60%)	6 (40%)	0	0
Thrombocytopenia	11 (33%)	4 (36%)	3 (27%)	2 (18%)	2 (18%)
Leukopenia	11 (33%)	2 (18%)	6 (55%)	3 (27%)	0
Hyperglycemia	9 (27%)	8 (89%)	0	1 (11%)	0

### Efficacy

**Fig. 3:**
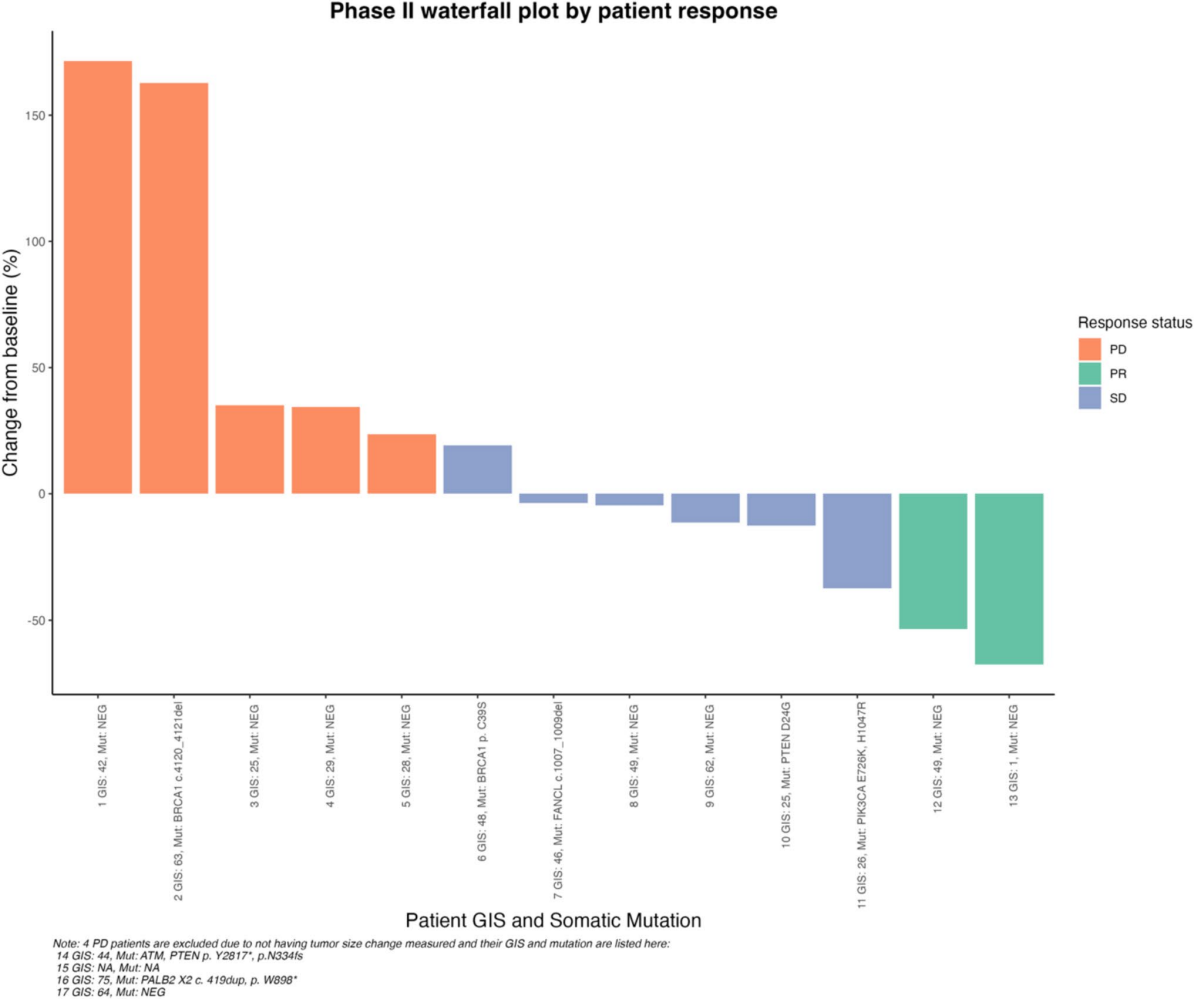
Patient response by GIS and somatic mutation status

The ORR in the phase II cohort (excluding the 2 patients with a gBRCA2 m) was 12% (95% CI 0.01, 0.36) with 2/17 patients achieving PR. Of those 2 patients, one had a duration of response of 1.9 months and the other of 8 months. An additional 6 patients (35%) attained SD, with a CBR at 16 weeks of 23.5%. The remaining 9 patients (53%) had a best response of PD (Fig. 3). Four of those 9 patients experienced clinical progression prior to first imaging assessment, two with physical exam findings (enlarging cervical and axillary lymph nodes, respectively), one with lab results consistent with hepatic progression, and one with a new brain metastasis. There were 6 patients who had received prior carboplatin in the metastatic setting. None of those 6 patients had a response to the investigational treatment. Three experienced a best response of SD, and the other 3 had a best response of PD. The median duration of study therapy was 1.6 months (range 0.6–7.35 months).

**Fig. 4:**
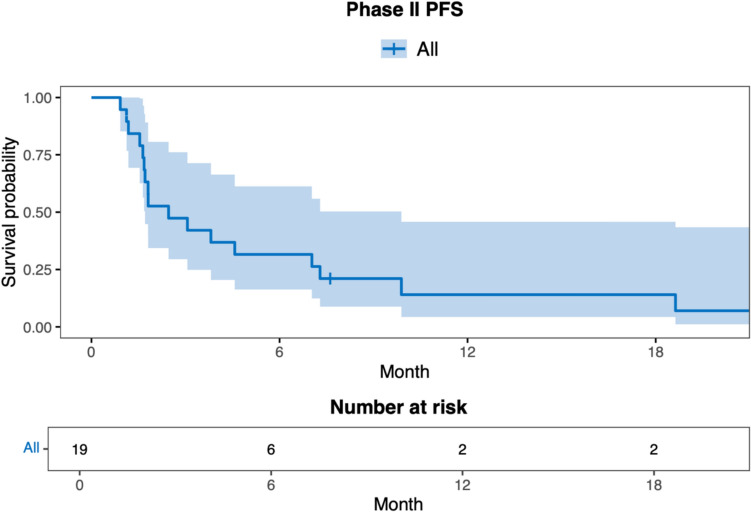
Progression-free survival in all phase II patients

**Fig. 5:**
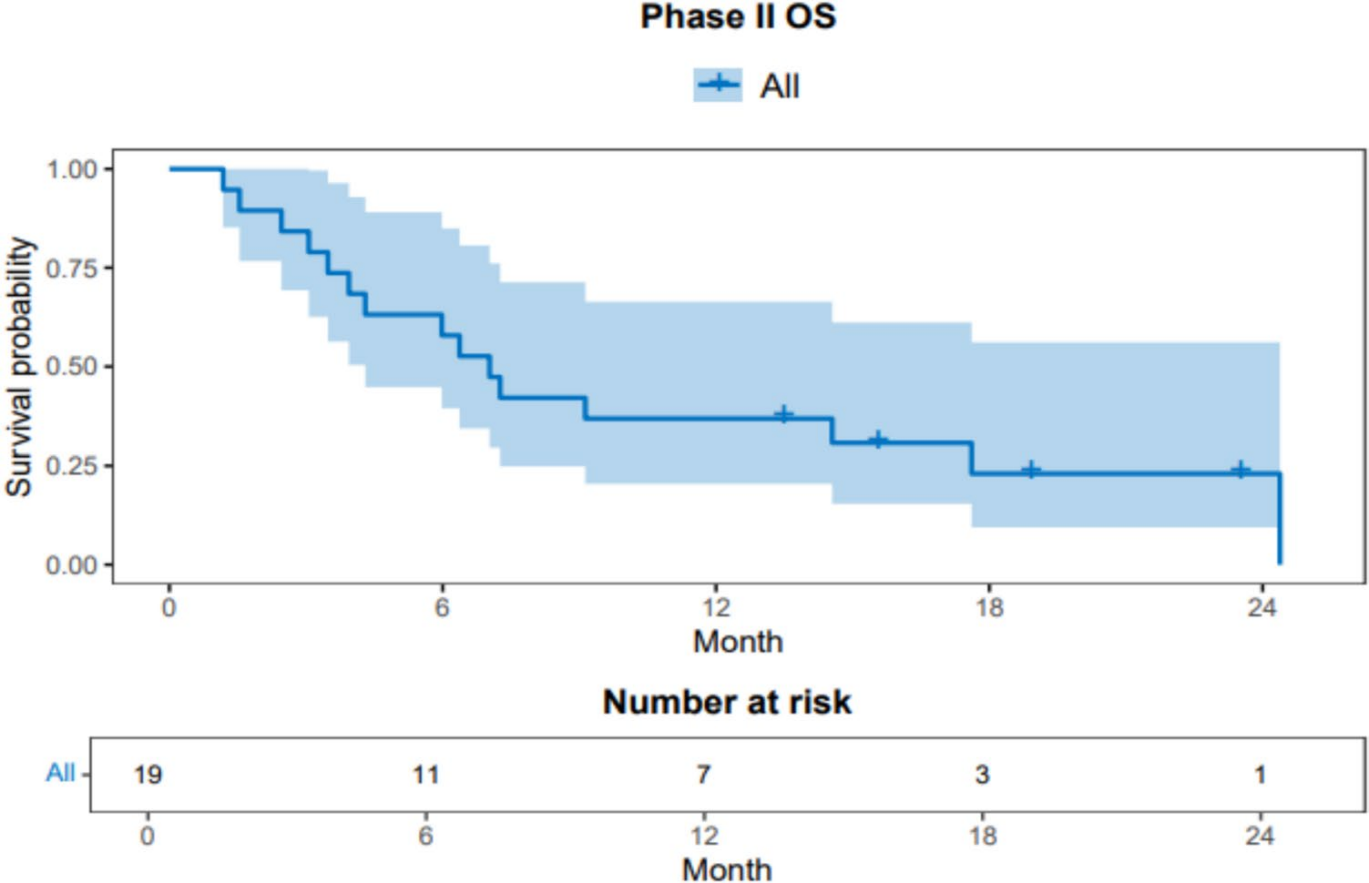
Overall survival in all phase II patients

Including all phase II patients, the median PFS was approximately 2.5 months (95% CI 1.71, 9.89) and median OS was approximately 7 months (95% CI 4.3, NA). The upper bound of median OS 95% CI was not estimable due to limited follow-up duration. Figures 4 and 5 demonstrate survival endpoints. One patient with a gBRCA2 m and ER + HER2- disease, treated at dose level 2 of the dose escalation experienced a CR of target lesions (overall PR) and was on study therapy for 38 months until PD. All 5 patients (including phase I) with a gBRCA1/2 m achieved a best response of either PR (3) or SD (2).

### Correlatives

Using the Myriad HRD assay, patient tumor samples from Phase II were examined. Response was analyzed by GIS, using a cutoff of ≥ 33 as high GIS (Fig. 3 and Table S3 in Supplementary). Patients with a known gBRCA1/2 m were not included in this analysis, and 1 patient did not have available tissue at the time of analysis. Fisher exact test was performed, and there was no statistically significant difference in response (PR or CBR16) based on GIS (*p* = 0.63).

The Myriad assay also analyzed clinically significant mutations by next-generation sequencing. Of the 17 patients in the phase II cohort, 6 had a clinically significant mutation (genes involved in DNA repair or in the PI3 K pathway). All of these 6 had a high GIS. Two patients had a sBRCA1 m (without corresponding germline mutation), with 1 experiencing best response of SD and the other PD. Of the 2 patients with a partial response, one had a high GIS (49) and no somatic mutations identified and the other a low GIS (1) with a TP53 mutation and MYC amplification, found on next-generation sequencing analysis done for clinical care purposes. Best responses in patients with a clinically significant deleterious somatic mutation are detailed in Fig. 3 and Table S4 in the Supplementary.

## Discussion

This study showed that while the combination of talazoparib and gedatolisib can be given safely at full doses, the combination did not meet the predefined efficacy threshold for ORR with only 12% of patients with advanced TNBC without a gBRCA1/2 m experiencing a response. The patients with advanced TNBC enrolled in this trial demonstrated a short PFS and OS, likely as they were pretreated with 1–3 lines of standard therapies and had limited treatment options beyond this study.

The clinical benefit rate at 16 weeks (CBR16) was 23%, suggesting there may be a population of patients with TNBC who could experience a chemotherapy-free period of treatment with this approach. However, a biomarker to prospectively identify this cohort was not identified with either the Myriad HRD score or sBRCA1/2 m. The results of this trial do not support continued investigation of this combination in all-comers with mTNBC. One patient with a gBRCA2 mutation remained on therapy for 38 months, far surpassing the median OS in the EMBRACA trial of approximately 19 months. Although preclinical data suggests that PI3 K inhibition may augment the effect of PARPi, this study did not have the sample size to address this question.

In our study, 4 patients (12%) developed pneumonitis, with 2 experiencing grade 3 pneumonitis. Trial eligibility criteria excluded patients with a history of drug-induced pneumonitis within the prior 12 months, those with a history of pneumonitis related to an mTOR inhibitor, and those with clinically significant pulmonary disease unrelated to breast cancer, thus the enrolled cohort of patients were not at particularly high risk of developing drug-related pneumonitis. This is a well-known side effect of mTOR inhibitors such as everolimus, with pneumonitis seen in both the BOLERO-2 trial (3%) and PrE0102 trial (17% overall, 6% grade 3) [24, 25]. While less common, pneumonitis has also been reported with PI3 K inhibitors [26]. Given that gedatolisib inhibits both PI3 K and mTOR, the side effect profile and risk of pneumonitis may be less favorable. Going forward, if a similar targeted combination is tested in another study, a PI3 K-specific targeted agent may be a better choice for evaluating the effect of PI3 K inhibition with less potential for pneumonitis, since the rates of pneumonitis appear to be lower with PI3 K inhibitors than mTOR inhibitors [24, 27].

Our study was the first to combine a PARPi with an mTOR/PI3 Ki in patients with mTNBC. Several studies, mostly pre-clinical, have evaluated the use of HRD status as a method of measuring “BRCA-ness” and thus tried to predict response to treatments such as PARPi. Our study was also the first clinical trial that we know of evaluating HRD as a predictive biomarker in wildtype mTNBC patients treated with PARPi. HRD as measured by GIS was not correlated with response in our study, although the cohort was small, there were very few responses, and it was not powered to detect a difference. Results of our study would suggest caution in using HRD status as a predictive biomarker in a clinical setting at this time. Functional HRD assessments are being developed and will need to be tested to determine if these will be better predictors of agents targeting HRD in tumors [28].

In addition to enrolling a small cohort of patients, this study had other limitations. Our patient population was pre-treated with chemotherapy, including carboplatin in some patients. This could have influenced both the safety outcomes and generalizability of the results to broader populations, especially with the potential cross-resistance between carboplatin and PARPi [29, 30]. Furthermore, 4 patients enrolled in this trial progressed clinically prior to their first imaging assessment, highlighting the challenges with developing novel therapy for an aggressive disease.

In conclusion, this chemotherapy-free approach to treatment in patients with pre-treated mTNBC did not meet its primary efficacy response threshold for continued investigation, however, it was safe with mostly low-grade TEAEs. Further pre-clinical studies should be considered to improve our understanding of this targeted therapy combination and if future clinical studies are warranted.

## Supplementary Information

Below is the link to the electronic supplementary material.

Supplementary file1 (DOCX 16 KB)

## Data Availability

The datasets generated during and/or analyzed during the current study are not publicly available due to patient privacy and to comply with data use agreements, but de-identified datasets may be available from the corresponding author on reasonable request.
