# Supplementary material for: Phase I/II trial investigating gedatolisib plus talazoparib in advanced triple negative or BRCA1/2 positive, HER2 negative breast cancers
Source: Breast Cancer Res Treat. 2025 Jun 5;212(3):521–30. doi: 10.1007/s10549-025-07747-x (PMC12208992; doi:10.1007/s10549-025-07747-x)
Supplement: Supplementary file 1 — Supplementary file1 (DOCX 16 KB) [file 10549_2025_7747_MOESM1_ESM.docx]

Supplementary

Table S1 Dose levels of Gedatolisib and Talazoparib in Phase I Safety Run-in

| **Dose Level** | **Gedatolisib** | **Talazoparib** |
| --- | --- | --- |
| -1 | 150 mg IV Days 1,8,15, and 22 | 0.75 mg orally qd Days 1-28 |
| 1* | 180 mg IV Days 1,8,15, and 22 | 0.75 mg orally qd Days 1-28 |
| 2 | 180 mg IV Days 1,8,15, and 22 | 1 mg orally qd Days 1-28 |
| *starting dose level | | |

Table S2 Overall Response in Phase II

| **Best Response** | **Number of patients** |
| --- | --- |
| Complete Response | 0 |
| Partial Response | 2 |
| Stable Disease | 6 |
| Progressive Disease | 9 |

Table S3 Response by Genomic Instability Score

| **Genomic Instability Score (GIS)** | **GIS high (≥33)** | | **GIS low (<33)** | |
| --- | --- | --- | --- | --- |
| Number of patient samples (N=17) | 10 (63%) | | 6 (37%) | |
| Best response by RECIST 1.1 | PD | 5 (50%) | PD | 2 (33%) |
|  | PR or SD | 5 (50%) | PR or SD | 4 (67%) |

*Analysis by Fisher exact test, p=0.63, no significant difference in response between GIS groups

Table S4 Deleterious/suspected deleterious somatic mutations in DNA repair genes or in PI3K pathway identified in individual patients from Phase II

| **Gene** | **Mutation** | **Best response** |
| --- | --- | --- |
| *BRCA1* | c.4120_4121del | PD |
| *BRCA1* | p. C39S | SD |
| *ATM* | p. Y2817* | PD |
| *PALB2* | c. 419dup  p. W898* | PD |
| *FANCL* | c.1007_1009del | SD |
| *PTEN* | p. D24G | SD |
| *PTEN* | p.N334fs | PD |
| *PIK3CA* | p. H1047R  p. E726K | SD |
